# Clinical observations of bone marrow transfusion for promoting bone marrow reconstruction after chemotherapy for AIDS-related lymphoma

**DOI:** 10.1186/s12865-021-00399-8

**Published:** 2021-01-28

**Authors:** Yixuan Liu, Suhong Xie, Lei Li, Yanhui Si, Weiwei Zhang, Xin Liu, Lin Guo, Baochi Liu, Renquan Lu

**Affiliations:** 1 Department of Clinical Laboratory, Fudan University Shanghai Cancer Center, No.270, Dong’An Road, Xuhui District, Shanghai, 200032 China; 2 Department of Oncology, Shanghai Medical College, Fudan University, Shanghai, China; 3 Department of surgery, Shanghai Public Health Clinical Center, Affiliated to Fudan University, Shanghai, China

**Keywords:** AIDS-related lymphoma, Autologous bone marrow transfusion, Chemotherapy, Bone marrow reconstruction

## Abstract

**Background:**

This study investigates the effect of autologous bone marrow transfusion (BMT) on the reconstruction of both bone marrow and the immune system in patients with AIDS-related lymphoma (ARL).

**Methods:**

A total of 32 patients with ARL participated in this study. Among them, 16 participants were treated with conventional surgery and chemotherapy (control group) and the remaining 16 patients were treated with chemotherapy followed by autologous bone marrow transfusion via a mesenteric vein (8 patients, ABM-MVI group) or a peripheral vein (8 patients, ABM-PI group). Subsequently, peripheral blood and lymphocyte data subsets were detected and documented in all patients.

**Results:**

Before chemotherapy, no significant difference in indicators was observed between three groups of ARL patients. Unexpectedly, 2 weeks after the end of 6 courses of chemotherapy, the ABM-MVI group, and the ABM-PI group yielded an increased level of CD8^+^T lymphocytes, white blood cells (WBC), and platelet (PLT) in peripheral blood in comparison to the control group. Notably, the number of CD4^+^T lymphocytes in the ABM-PI group was significantly higher than that in the other two groups. Additionally, no significant difference in haemoglobin levels was observed before and after chemotherapy in both the ABM-MVI and ABM-PI groups, while haemoglobin levels in the control group decreased significantly following chemotherapy.

**Conclusions:**

Autologous bone marrow transfusion after chemotherapy can promote the reconstruction of both bone marrow and the immune system. There was no significant difference in bone marrow recovery and reconstruction between the mesenteric vein transfusion group and the peripheral vein transfusion group.

**Supplementary Information:**

The online version contains supplementary material available at 10.1186/s12865-021-00399-8.

## Background

AIDS, the most common cause of morbidity and mortality globally, causes more than 1.1 million deaths annually of individuals mostly older than 15 years old. With the introduction of antiretroviral therapy, serious non-AIDS complications (for example malignancies) have occurred, which have a mortality risk twice that of AIDS-related events [1, 2]. AIDS-Related Lymphoma (ARL) is a common malignancy in the HIV-infected population [3]. The risk of developing lymphoma steadily increases with an increasing duration of HIV infection and advancing immunosuppression [4, 5].

Patients who develop ARL are generally reactive to chemotherapy, with some reportedly cured by chemotherapy [6]. However, the chemotherapy was myelotoxic, and 75 and 52% of patients experienced grade 4 neutropenia and thrombocytopenia respectively. The treatment-related mortality rate is 10% [7]. Additionally, chemotherapeutic drugs can cause organ damage and bone marrow suppression, and potentially causing even more severe immune damage to people with AIDS [8]. Therefore, bone marrow suppression should be addressed for patients being treated with chemotherapy, especially patients whose immune system is compromised as a result of their disease. It is essential to refine the investigational approaches to ARL treatment because the majority of patients present with advanced AIDS and poor prognostic factors, while less than one third have no adverse prognostic factors and reasonable prospects for long-term survival.

Previously, we have investigated the same treatment regimen on patients with AIDS and decompensated liver cirrhosis [9]. We conducted infusion-port intubation through the right omental vein after splenectomy. The patient’s autologous bone marrow was collected and then transfused into the portal vein through the infusion-port. The findings confirmed that autologous bone marrow transfusion can promote liver function reconstruction without any side effects.

To investigate the effect of autologous bone marrow transfusion on the reconstruction of both bone marrow and the immune system in patients with ARL, we undertook a clinical study investigating 32 patients with ARL between March 2019 and March 2020. These patients were considered in three groups: a control group, the ABM-MVI group and the ABM-PI group. Following treatment, peripheral blood and lymphocyte diagnostic subsets were detected and documented in all patients. Before chemotherapy, each patients’ autologous bone marrow was collected and stored at − 20 °C in a preservation solution. Five days after chemotherapy, those patients with bone marrow preservation were administered the ABM via either an infusion-port or a peripheral vein. In comparison with patients that received conventional chemotherapy, the levels of white blood cells (WBC), platelets (PLT), and haemoglobin (HB) in patients treated with an autologous bone marrow transfusion after chemotherapy were significantly increased (*P* < 0.05). These findings, following an autologous bone marrow transfusion, indicate that the stem cells in bone marrow significantly promotes the reconstruction of bone marrow. Additionally, our data support the finding that autologous bone marrow transfusion after chemotherapy does promote the reconstruction of both the bone marrow and immune systems. There was no significant difference in the bone marrow recovery and reconstruction outcomes between those patients in the mesenteric vein transfusion group and peripheral vein transfusion group.

Our research demonstrates that autologous bone marrow transfusion can promote the reconstruction of bone marrow and the immune system following chemotherapy. These results provide insight so that better protocols and strategies can be developed for the treatment of AIDS-related diseases.

## Results

### Patients disposition

**Fig. 1:**
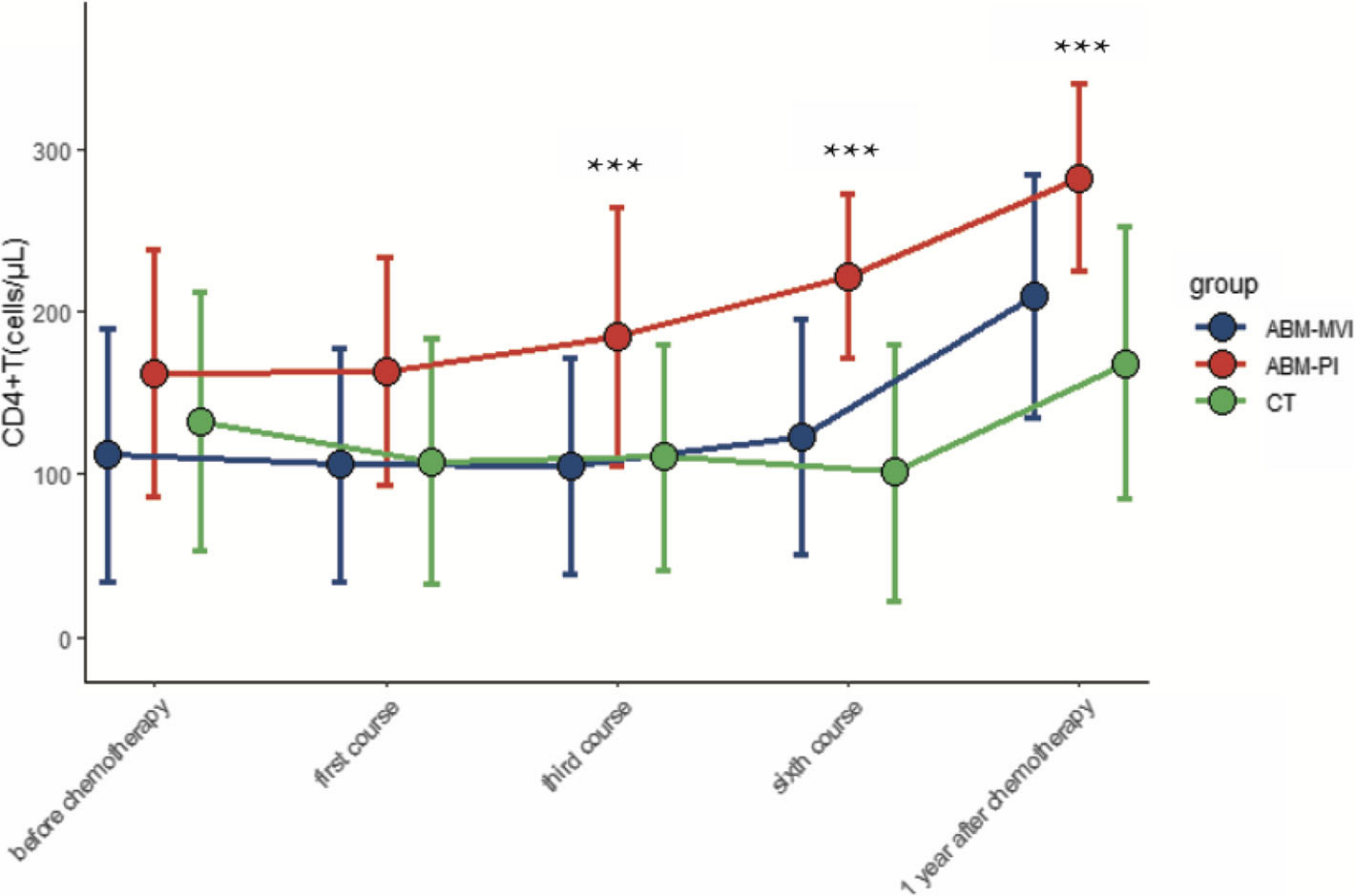
Changes of CD4^+^T value in the three groups. The number of CD4 + T cells in the ABM-PI group was significantly higher than that in the CT group and ABM-MVI group during the third and sixth courses of chemotherapy, as well as the one year post-chemotherapy time point

**Fig. 2:**
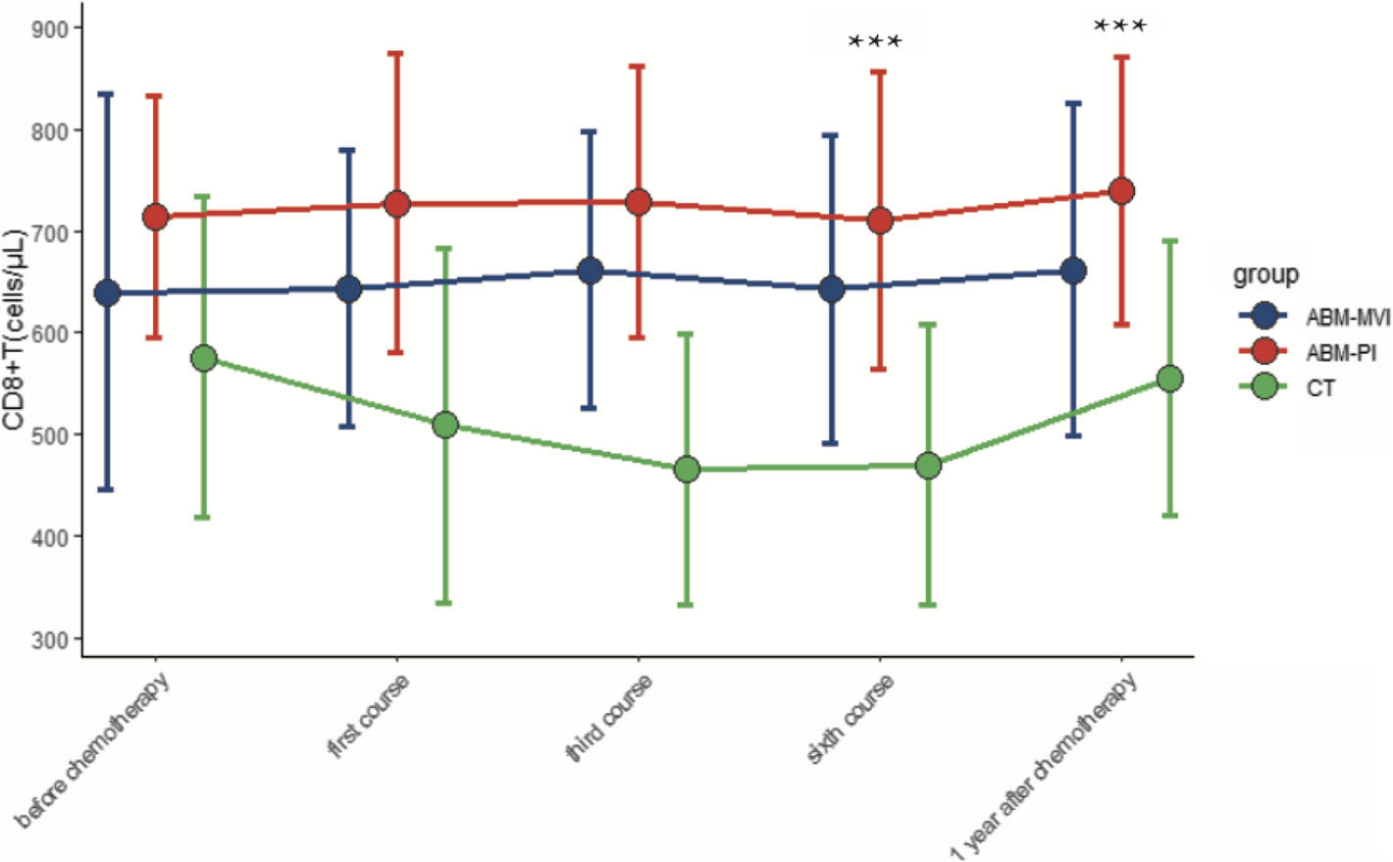
Changes of CD8^+^T value in the three groups. The number of CD8 + T cells in the ABM-MVI group and the ABM-PI group were significantly higher than in the CT group at each time point of the study

**Fig. 3:**
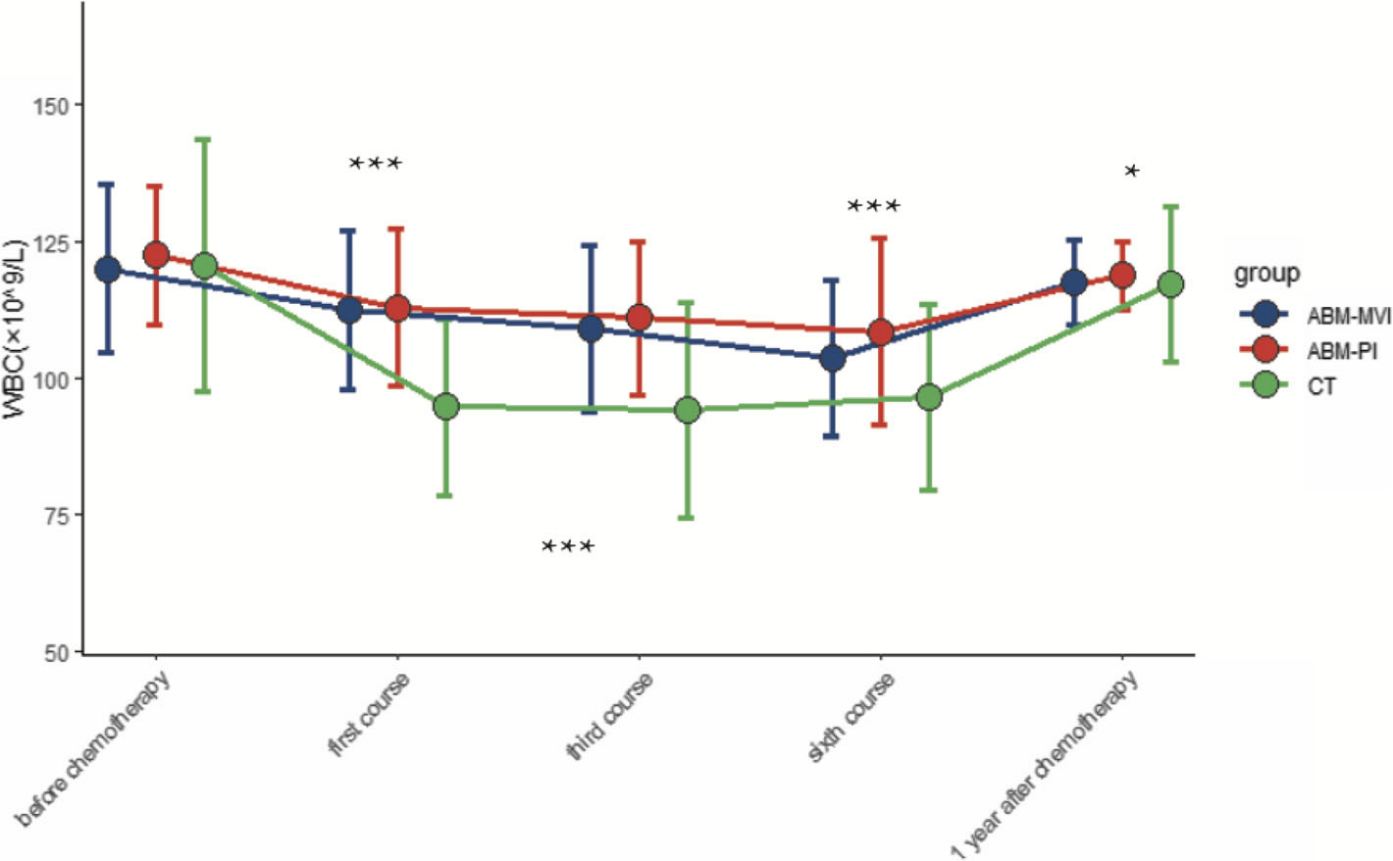
Changes of white blood cells value in the three groups. The number of WBC in the ABM-MVI group and the ABM-PI group were significantly higher than in the CT group at each time point of the study after transfusion

**Fig. 4:**
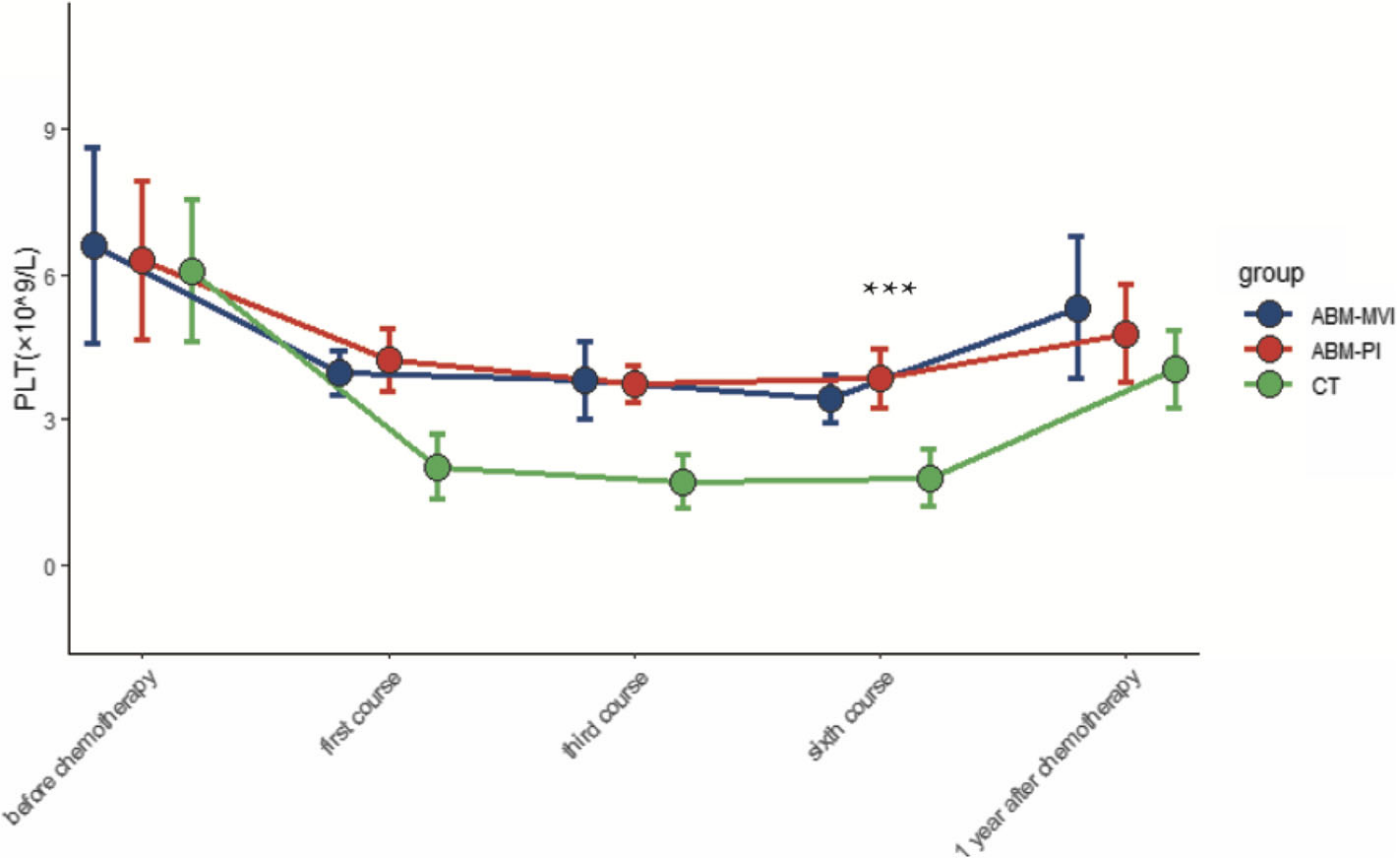
Changes of platelets value in the three groups. Before chemotherapy, during the first course, the third course, the sixth course, and one year after the chemotherapy, the level of PLT in each patient group first decreased and then increased. In the sixth course, the level of PLT in the ABM-MVI and the ABM-PI groups were significantly higher than that in the CT group

**Fig. 5:**
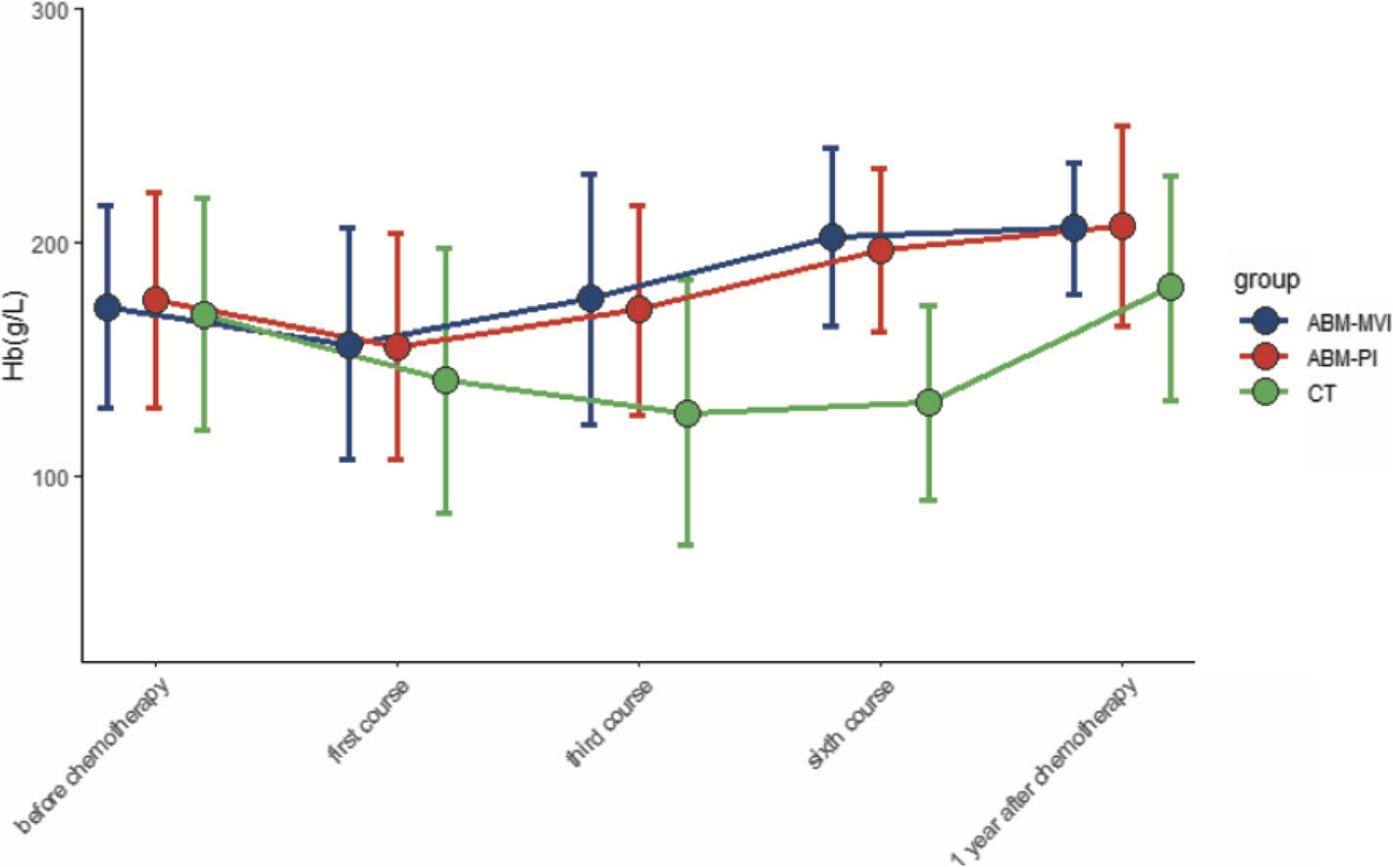
Changes of haemoglobin value in the three groups. There was no significant difference in HB levels between all three groups before chemotherapy and following chemotherapy, the HB levels in each group showed a downward trend. Compared with the CT group, the decline in HB levels in both the ABM-MVI and the ABM-PI groups was more moderate

There were 32 patients (19 males and 13 females) involved in this study, aged between 22 and 70 years of age. All the surgical procedures undertaken went smoothly, without any complications. The control group of 16 participants was treated using conventional surgery and chemotherapy, while the remaining 16 patients were treated with chemotherapy followed by an autologous bone marrow transfusion via a mesenteric vein (ABM-MVI group) or a peripheral vein (ABM-PI group). Subsequently, peripheral blood and lymphocyte diagnostic subsets were detected and documented for all patients. Postoperative histopathological examination showed that 17 cases had a diffuse large B lymphoma, 12 had a Burkitt lymphoma, and 3 were other types of lymphoma. Patients’ PET-CT imaging results were normal, with no bone metastases detected. Chemotherapy was then started once the wound’s healing was practically complete at day 14 postoperative. Data from the three groups (CT group, ABM-MVI group, and ABM-PI group), with or without bone marrow transfusion, showing the levels of CD4 + T lymphocytes, CD8 + T lymphocytes, white blood cells (WBC), platelets (PLT), and haemoglobin (HB) at different times are shown in Figs. 1, 2, 3, 4 and 5.

### Reconstruction of the immune system

**Fig. 6:**
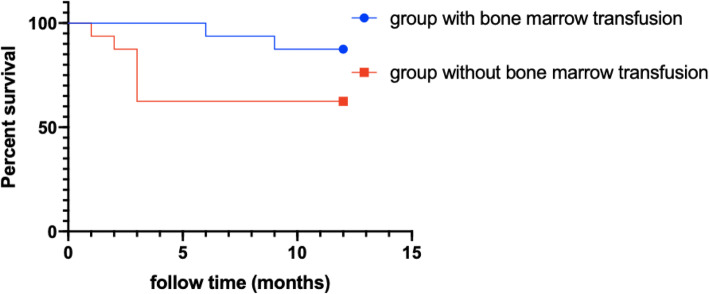
Survival plot between patients with bone marrow transfusion and without bone marrow transfusion. However, the difference was not statistically significant (*P* > 0.05) because of the insufficient number of participants

In the bone marrow transfusion groups, two patients died of tumour metastasis within 1 year postoperative. In the group without bone marrow transfusion, 6 patients died of infection or tumour metastasis within 1 year after the operation. There were compelling differences between the two treatment groups (with or without ABM), however, the difference was not statistically significant (*P* > 0.05, Fig. 6) because of the insufficient number of participants. In the ABM-PI group, the number of CD4 + T cells was significantly higher than in the CT group and ABM-MVI group during the third and sixth courses of chemotherapy, as well as the 1 year post-chemotherapy time point. There was no statistical difference in the number of CD4^+^T lymphocytes between the three groups before treatment (*P* > 0.05). After treatment, however, each group showed a gradual upward trend. In the third course, the sixth course and 1 year after chemotherapy, the elevated CD4^+^T lymphocytes level of the ABM-PI group was greater than in both the ABM-MVI and the CT groups, and the difference was found to be statistically significant (*P* < 0.05, see Table 1). The number of CD8 + T cells in the ABM-MVI group and the ABM-PI group were significantly higher than in the CT group at each time point of the study (P < 0.05, see Table 2).

**Table 1:** Comparison of changes in CD4^+^T lymphocytes (cells/μl) after bone marrow transfusion vs without bone marrow transfusion

Group	before chemotherapy	first course	third course	sixth course	1 year after chemotherapy
CT	132.31 ± 79.88	107.81 ± 75.34	110.81 ± 69.38	101.08 ± 78.61	168.20 ± 83.66
ABM-MVI	112.00 ± 77.69	105.63 ± 72.29	105.25 ± 66.71	122.75 ± 72.38	209.43 ± 74.17
ABM-PI p-value	162.50 ± 75.87 0.475	163.13 ± 70.14 0.149	184.63 ± 79.78^ab^ 0.04	221.75 ± 50.10^ab^ 0.001	282.14 ± 57.76^ab^ 0.004

p-value: one-way ANOVA (P < 0.05 means there was statistically significant in the three groups)

a: the difference was statistically significant between ABM groups (ABM-MVI or ABM-PI)

and CT group (^a^*P* < 0.05); b: the difference was statistically significant between the two ABM groups, (^b^P < 0.05)

**Table 2:** Comparison of changes in CD8^+^T lymphocytes (cells/μl) after bone marrow transfusion vs without bone marrow transfusion

Group	before chemotherapy	first course	third course	sixth course	1 year after chemotherapy
CT	575.81 ± 158.76	509.25 ± 174.40	466.81 ± 133.08	470.08 ± 137.31	555.40 ± 134.53
ABM-MVI	639.75 ± 194.12	643.63 ± 135.72^a^	661.63 ± 135.38^a^	643.50 ± 151.18	661.43 ± 162.91
ABM-PI p-value	713.25 ± 117.96^a^ 0.042	726.88 ± 146.54^a^ 0.007	728.38 ± 133.67^a^ 0.001	709.88 ± 145.79 0.001	738.71 ± 130.77 0.11

p-value: one-way ANOVA

Compared with CT group, ^a^P < 0.05; compared with ABM-MVI group, ^b^P < 0.05

### Numbers of WBC, PLT, HB were significantly increased after transfusion

Before chemotherapy, during the first course, the third course, the sixth course, and 1 year after the chemotherapy, the level of white blood cells (WBC) and platelets (PLT) in each patient group first decreased and then increased (Additional file: Table S1–2). The level of PLT in the ABM-MVI and the ABM-PI groups were significantly higher than that in the CT group in the sixth course (*P* < 0.05). And the levels of WBC in the ABM-MVI and the ABM-PI groups were significantly higher than those in the CT group in each time point. There was no significant difference in haemoglobin levels between all three groups before chemotherapy (*P* > 0.05) and following chemotherapy, the haemoglobin levels in each group showed a downward trend. Compared with the CT group, the decline in haemoglobin levels in both the ABM-MVI and the ABM-PI groups was more moderate (*P* < 0.05) (Table 3).

**Table 3:** Comparison of changes in HB (g/L) after bone marrow transfusion vs without bone marrow transfusion

Group	before chemotherapy	first course	third course	sixth course	1 year after chemotherapy
CT	120.56 ± 23.17	94.63 ± 16.05	94.06 ± 19.62	96.54 ± 16.99	117.10 ± 14.18
ABM-MVI	120.00 ± 15.36	112.38 ± 14.55^a^	109.00 ± 15.22^a^	103.63 ± 14.23	117.43 ± 7.74
ABM-PI p-value	122.50 ± 12.66 0.978	112.88 ± 14.29^a^ 0.006	110.88 ± 13.98 0.036	108.50 ± 17.12 0.209	118.71 ± 6.18 0.937

p-value: one-way ANOVA

Compared with CT group, ^a^P < 0.05; compared with ABM-MVI group, ^b^P < 0.05

### There was no significant difference between the two methods of transfusion

Compared with the CT group, autologous bone marrow transfusion increased the patients’ bone marrow reconstruction. Comparison of the outcomes of the two different bone marrow transfusion methods (either portal vein or peripheral vein), shows that the number of CD4 + T cells in the PI group was significantly higher than in MVI groups during the third and sixth courses of chemotherapy as well as at the 1 year after chemotherapy timepoint. However, no significant difference in bone marrow recovery and reconstruction was observed in comparisons between the mesenteric vein transfusion group and the peripheral vein transfusion group. It is more convenient and less intrusive to inject autologous bone marrow via a peripheral vein.

## Discussion

### The treatment of ARL is currently a challenge, autologous bone marrow transfusion brings hope

Patients have bone marrow suppression and increased immune system damage after the second course of conventional chemotherapy treatment and need to extend the intervals between recovery. In this study, each patients’ autologous bone marrow was preserved before chemotherapy and administered via transfusion after chemotherapy. The results of this study show that autologous bone marrow transfusion can significantly promote patients’ bone marrow reconstruction and recovery [10].

AIDS is the most common cause of morbidity and mortality worldwide. ARL is one of the common malignancies in the HIV-infected population, a cohort who generally have significant impairment of their immune function [11, 12]. The number of CD4^+^T cells reported for healthy people is above 500 cell/μl [13]. However, the average levels of CD4^+^T lymphocytes in the 32 participants in this study were below 200 cell/μl. Generally, chemotherapy is used as the first-line treatment for patients with lymphoma. However, chemotherapy can cause organ damage and bone marrow suppression, and may even cause more severe immune damage to HIV-infected people [14]. In this study, we investigated the transfusion of an autologous bone marrow sample as an adjuvant treatment for chemotherapy-induced myelosuppression. The results suggested that autologous bone marrow transfusion is effective in protecting CD4^+^T, CD8 + T, WBC, PLT, HB levels in AIDS patients who require treatment for concomitant ARLs.

### There was no significant difference in results between the two transfusion groups

Chemotherapy is the conventional treatment regimen of ARL, but chemotherapy is cytotoxic to both bone marrow and lymphocytes [15]. These associated side effects can limit the doses and length of chemotherapy treatments that can be administered. In the present study, we added autologous bone marrow transfusions to the conventional treatment regimen. As a direct consequence, the fatality rate of the conventional chemotherapy group within 1 year is 35.5%, while that of the autologous bone marrow preservation transfusion group is 12.5%. Statistically, however, no significant difference was attributable (*P* > 0.05), presumably due to the small number of participants in this study. Surprisingly, the data show that autologous bone marrow transfusions after chemotherapy could promote the reconstruction of bone marrow and the immune system. Additionally, no significant difference in bone marrow recovery and reconstruction between the mesenteric vein transfusion group and the peripheral vein transfusion group was observed.

### Autologous bone marrow transfusion is effective in protecting the immune system

The administration of antiretroviral drugs to HIV patients can control the destruction of CD4^+^T lymphocytes and promote immune reconstruction [16]. Patients were receiving antiretroviral treatment at the same time as chemotherapy. Although chemotherapy can reduce levels of CD4^+^T lymphocytes for a short time, the number of CD4^+^T gradually increases following an autologous bone marrow transfusion [17].

In conclusion, an autologous bone marrow transfusion after chemotherapy could promote the reconstruction of both bone marrow and the immune system. There was no significant difference in the observed bone marrow recovery and reconstruction between the mesenteric vein transfusion group and the peripheral vein transfusion group. The major limitations of this study were the relatively modest sample size and the limited clinical information.

## Conclusion

Our institutions are currently enrolling more participants so that future studies will have an improved clinical approach and a larger number of participants so that more statistically valid observations and conclusions can be determined.

## Methods

### Patients

The study participants were a total of 32 ARL patients hospitalised in our centre between March 2019 and March 2002. The study group was comprised of 19 males and 13 females, aged between 22 and 70 years old. All the patients had been treated with tumour resection or biopsy. All the patients’ pathological results showed that the lesions were non-Hodgkin’s lymphoma. Detection of HIV antibodies was undertaken before surgery, and the HIV infection was confirmed at the local CDC. All patients have been treated with antiretroviral drugs.

From this patient cohort, 16 participants were treated with conventional surgery and chemotherapy (control group), while another 16 patients were treated with chemotherapy followed by autologous bone marrow transfusion via either a mesenteric vein (ABM-MVI group) or a peripheral vein (ABM-PI group). Measurements of the peripheral blood and lymphocyte diagnostic subsets were undertaken before and after chemotherapy.

### Treatment

All patients were treated with antiretroviral therapy before surgery. Any additional health issues and/or diseases detected with the 32 participants were also treated before the study commenced, such as anti-tuberculosis, antifungal, and other supportive treatments. The total number of white blood cells (WBC) of patients was often lower than 2 × 10^9^ / L, so granulocyte stimulating factors or blood transfusions were administered to patients with severe anaemia. Patients with neck cancer, axillary cancer or inguinal cancer had either the surgical resection or a biopsy of the tumours, and excised specimens were analysed by pathologists. Open surgery was conducted to remove tumours in patients with abdominal cancer, and they were monitored for postoperative complications such as intestinal obstruction and gastrointestinal bleeding. An infusion-port intubation through the right omental vein was undertaken for 8 patients. The doses and schedules of chemotherapy regimens were determined for each patient according to the pathological staging of the detected tumours.

CHOP-based Chemotherapy was administered to all 32 patients [18]: CTX (Cyclophosphamide) 750 mg/m^2^ IV day 1, ADM (Adriamycin) 500 mg/m^2^ IV day 1, VCR (Vincristine, Oncovin) 1.4 mg/m^2^ IV day 1, Pred (Prednisone) 100 mg PO day 1 to day 5, or add Rituximab (R-CHOP) 375 mg/m^2^ IV day 1. Repeat every 3 weeks for 6 cycles.

### Bone marrow collection and transfusion

Approximately 3 weeks after surgery, the bone marrow of each patient was collected before the commencement of chemotherapy treatment. Patients, lying in a supine position and under local anaesthesia, were punctured with a bone marrow biopsy needle under sterile conditions at the right or left anterior superior iliac crest. Bone marrow was collected via a special porous bone marrow collector for 50 ml. The bone marrow samples were then injected into a 100 ml plastic bag containing 50 ml of bone marrow preservation solution and stored at − 20 °C. Following 5 days of chemotherapy, the stored bone marrow was placed in a water bath at 39 °C and kept swinging for 3 min to rewarm. The bone marrow was then infused into the patient’s portal vein via the infusion-port. The abdomen was sterilized before infusion and the infusion-port rinsed and closed with normal saline. Alternatively, the bone marrow was transfused via the patients’ peripheral vein.

### Statistical analyses

SPSS 16.0 statistical software was used for analysis. Measurement data were expressed as mean ± standard deviation ($\overline{x}$ ±s), and *P* < 0.05 was considered statistically significant. The One-way ANOVA was used to describe the *p*-value in tables.

Peripheral blood and lymphocyte subsets were determined before chemotherapy. After 5 days of chemotherapy, an autologous bone marrow transfusion was undertaken. After 10 days of chemotherapy (it was also the 5 days of autologous bone marrow transfusion), routine blood and lymphocyte data subsets were re-examined and recorded for control analysis.

## Supplementary Information

**Additional file 1.** Table S1. Comparison of changes in WBC (*10^9^/L) after bone marrow transfusion vs without bone marrow transfusion.

**Additional file 2.** Table S2. Comparison of changes in PLT (*10^9^/L) after bone marrow transfusion vs without bone marrow transfusion.

## Data Availability

All data generated or analyzed during this study are included in this published article and its supplementary information files.

## Abbreviations

BMT: bone marrow transfusion
ARL: AIDS-related lymphoma
ABM-MVI: autologous bone marrow transfusion via mesenteric vein
ABM-PI: autologous bone marrow transfusion via peripheral vein
WBC: white blood cells
PLT: platelet
HB: hemoglobin

## References

[1] Berry SA, Fleishman JA, Moore RD, Gebo KA (2012). Trends in reasons for hospitalization in a multisite United States cohort of persons living with HIV, 2001-2008. J Acquir Immune Defic Syndr.

[2] Neuhaus J, Angus B, Kowalska JD, La Rosa A, Sampson J, Wentworth D (2010). Risk of all-cause mortality associated with nonfatal AIDS and serious non-AIDS events among adults infected with HIV. Aids..

[3] Wang CC, Kaplan LD (2016). Clinical management of HIV-associated hematologic malignancies. Expert Rev Hematol.

[4] Joint United Nations Programme on HIV/AIDS (UNAIDS) and Joint United Nations Programme on HIV/AIDS (UNAIDS). Global AIDS Update 2016. Geneva; 2009. 12349391

[5] WHO (2015). Global health sector response to HIV, 2000–2015: focus on innovations in Africa.

[6] Apostolova N, Blas-García A, Esplugues JV (2011). Mitochondrial toxicity in HAART: an overview of in vitro evidence. Curr Pharm Des.

[7] Sparano JA, Lee S, Chen MG, Nazeer T, Einzig A, Ambinder RF (2004). Phase II trial of infusional cyclophosphamide, doxorubicin, and etoposide in patients with HIV-associated non-Hodgkin's lymphoma: an eastern cooperative oncology group trial (E1494). J Clin Oncol.

[8] Deeks SG (2011). HIV infection, inflammation, immunosenescence, and aging. Annu Rev Med.

[9] Liu B, Chen X, Wang Y, Shi Y (2013). Curative effect of hepatic portal venous administration of autologous bone marrow in AIDS patients with decompensated liver cirrhosis. Cell Death Dis.

[10] Kitchen SG, Zack JA (2011). Stem cell-based approaches to treating HIV infection. Curr Opin HIV AIDS.

[11] Brunnberg U, Hentrich M, Hoffmann C, Wolf T, Hubel K (2017). HIV-associated malignant lymphoma. Oncol Res Treat.

[12] Little RF (2017). Cancer clinical trials in persons with HIV infection. Curr Opin HIV AIDS.

[13] Viard JPBM, Hubert JB, Aaron L (2004). Impact of 5 years of maximally successful highly active antiretroviral therapy on CD4 cell count and HIV-1 DNA level. AIDS..

[14] Hauner K, Maisch P, Retz M (2017). Side effects of chemotherapy. Urologe A.

[15] Benicchi TGC, Cattaneo C, Casari S (2005). T-cell immune reconstitution after hematopoietic stem cell transplantation for HIV-associated lymphoma. Transplantation..

[16] Schommers P, Gillor D, Hentrich M, Wyen C, Wolf T, Oette M (2018). Incidence and risk factors for relapses in HIV-associated non-Hodgkin lymphoma as observed in the German HIV-related lymphoma cohort study. Haematologica..

[17] Michieli M, Mazzucato M, Tirelli U, De Paoli P (2011). Stem cell transplantation for lymphoma patients with HIV infection. Cell Transplant.

[18] Coiffier B, Lepage E, Brière J, Herbrecht R, Tilly H, Bouabdallah R (2002). CHOP chemotherapy plus rituximab compared with CHOP alone in elderly patients with diffuse large-B-cell lymphoma. N Engl J Med.

