# Supplementary material for: Clinical observations of bone marrow transfusion for promoting bone marrow reconstruction after chemotherapy for AIDS-related lymphoma
Source: BMC Immunol. 2021 Jan 28;22:10. doi: 10.1186/s12865-021-00399-8 (PMC7845098; doi:10.1186/s12865-021-00399-8)
Supplement: Supplementary file 1 — Additional file 1. Table S1. Comparison of changes in WBC (*109/L) after bone marrow transfusion vs without bone marrow transfusion. [file 12865_2021_399_MOESM1_ESM.docx]

| Group | before chemotherapy | first course | third course | sixth course | 1 year after chemotherapy |
| --- | --- | --- | --- | --- | --- |
| CT | 6.08±1.47 | 2.04±0.66 | 1.73±0.56 | 1.81±0.61 | 4.04±0.81 |
| ABM-MVI | 6.61±2.01^a^ | 3.96±0.46^a^ | 3.83±0.80^a^ | 3.44±0.51^a^ | 5.31±1.47^a^ |
| ABM-PI  p-value | 6.30±1.66  0.750 | 4.23±0.66^a^  <0.0001 | 3.73±0.38^a^  <0.0001 | 3.85±0.61^a^  <0.0001 | 4.79±1.03  0.019 |

p-value: one-way ANOVA

Compared with CT group, ^a^P <0.05; compared with ABM-MVI group, ^b^P <0.05
