# Supplementary material for: Clinical observations of bone marrow transfusion for promoting bone marrow reconstruction after chemotherapy for AIDS-related lymphoma
Source: BMC Immunol. 2021 Jan 28;22:10. doi: 10.1186/s12865-021-00399-8 (PMC7845098; doi:10.1186/s12865-021-00399-8)
Supplement: Supplementary file 2 — Additional file 2. Table S2. Comparison of changes in PLT (*109/L) after bone marrow transfusion vs without bone marrow transfusion. [file 12865_2021_399_MOESM2_ESM.docx]

| Group | before chemotherapy | first course | third course | sixth course | 1 year after chemotherapy |
| --- | --- | --- | --- | --- | --- |
| CT | 192.69.44±49.44 | 140.94±56.85 | 127.38 ±56.55 | 131.38±41.52 | 180.60±47.74 |
| ABM-MVI | 172.50±43.14 | 156.50±49.51 | 176.00±53.54 | 202.25±38.24^a^ | 206.14±28.38 |
| ABM-PI  p-value | 175.38±45.77  0.065 | 155.75±48.20  0.78 | 171.13 ±44.82  0.08 | 196.75±34.71^a^  <0.0001 | 207.29±42.91  0.267 |

p-value: one-way ANOVA

Compared with CT group, ^a^P <0.05; compared with ABM-MVI group, ^b^P <0.05
